# Tannins in Food: Insights into the Molecular Perception of Astringency and Bitter Taste

**DOI:** 10.3390/molecules25112590

**Published:** 2020-06-02

**Authors:** Susana Soares, Elsa Brandão, Carlos Guerreiro, Sónia Soares, Nuno Mateus, Victor de Freitas

**Affiliations:** REQUIMTE/LAQV, Faculdade de Ciências da Universidade do Porto, Rua do Campo Alegre, 689, 4169-007 Porto, Portugal; elsa.brandao@fc.up.pt (E.B.); up201911054@fc.up.pt (C.G.); sonia.soares@fc.up.pt (S.S.); nbmateus@fc.up.pt (N.M.)

**Keywords:** bitter taste receptors, mechanoreceptors, oral cells, polyphenols, salivary proteins, polysaccharides

## Abstract

Astringency and bitterness are organoleptic properties widely linked to tannin compounds. Due to their significance to food chemistry, the food industry, and to human nutrition and health, these tannins’ taste properties have been a line of worldwide research. In recent years, significant advances have been made in understanding the molecular perception of astringency pointing to the contribution of different oral key players. Regarding bitterness, several polyphenols have been identified has new agonists of these receptors. This review summarizes the last data about the knowledge of these taste properties perceived by tannins. Ultimately, tannins’ astringency and bitterness are hand-in-hand taste properties, and future studies should be adapted to understand how the proper perception of one taste could affect the perception of the other one.

## 1. Tannins Overview

Tannins are widely dispersed in the human diet due to the prevalent consumption of plant-based foods and beverages (e.g., red grapes, red wine, chocolate, tea, and beer) [1]. Several health benefits have been associated to these compounds, namely prevention of cancer, cardiovascular diseases, allergies, Alzheimer’s, and Parkinson’s disease [2]. Despite the continuous evolution of different techniques over the years, research groups still struggle to analyze tannins from foodstuffs since they remain very complex and many compounds can be easily overlooked [3]. Compound extraction, characterization of entire food matrices, and isolation/identification of compounds of high molecular weight are examples of the current challenges faced in this research area [4].

Tannins consist of a specific group of polyphenols that have the special ability to interact and precipitate alkaloids, gelatin, and other proteins [5]. This means that all tannins are polyphenols, but the reverse is not true.

**Figure 1:**
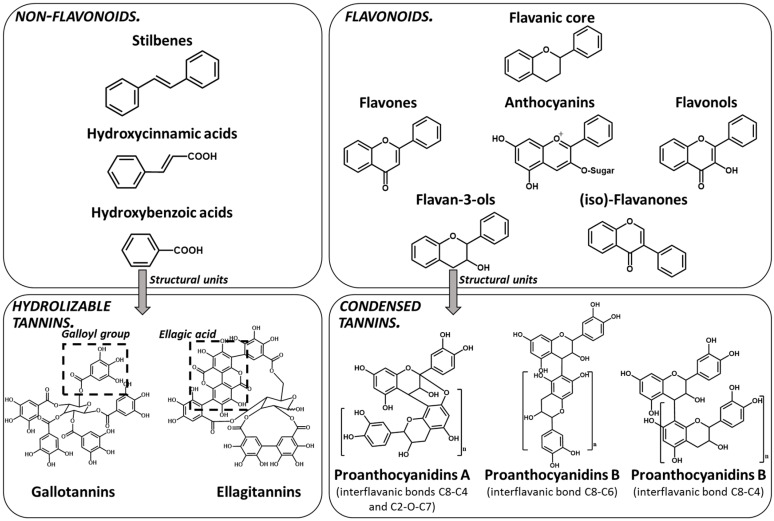
Phenolic compounds families: chemical structure core and classical division of some of the most common classes found in food.

Polyphenols are a large family of compounds that result from plant secondary metabolism as a chemical defense against predators and environmental aggressions. Polyphenols are classically divided in nonflavonoids (e.g., phenolic acids and stilbenes) and flavonoids (e.g., anthocyanins and flavan-3-ols). Among the most common dietary nonflavonoids are the phenolic acids, namely gallic acid, hydroxycinnamic acids and their conjugated derivatives, and stilbenes. Flavonoids present a typical C6-C3-C6 flavanic core (Figure 1) and are the most abundant of the polyphenols found widely spread through plant kingdom and therefore abundant in the human diet [6]. The main subgroups of the dietary flavonoids are flavones, anthocyanidins, flavonols, (iso)-flavanones, and flavan-3-ols (Figure 1).

The tannin group has a specific classification based on their molecular structure [1,7]. However, their building blocks are always derived from simpler polyphenols [1,8,9].

Tannins are subdivided in four different families: (1) condensed tannins (or proanthocyanidins); (2) hydrolyzable tannins; (3) phlorotannins (mainly present in brown algae); and (4) complex tannins. The condensed and hydrolyzable tannins are the two main families (Figure 1). Condensed tannin structure is essentially composed by units of the previously described flavan-3-ol units. Proanthocyanidins are composed by the diastereomers (−)-epicatechin/(+)-catechin subunits and/or by their galloylated derivatives. These subunits are linked through carbon–carbon linkages (interflavanic bond) that can occur between the carbon-4 of one unit and the carbon-8 of the adjacent unit (C4–C8) or between the carbon-4 of one unit and the carbon-6 of the adjacent unit (C4–C6). These interflavanic bonds occur in the proanthocyanidins type B. Proanthocyanidins type A have an additional ether-linkage at the carbon-2 of one unit and the carbon-7 (C2-O-C7) of the adjacent unit (Figure 1). Proanthocyanidins with a very large number of these structural units (up to about forty) have already been identified [10,11]. Besides a high diversity of mean degree of polymerization (mDP) and the type of interflavanic bonds between the units, condensed tannins also present different substitution patterns (galloylation and hydroxylation), contributing to a huge structural diversity of the condensed tannins in plants and foods.

Hydrolyzable tannins are further divided into ellagitannins and gallotannins [6]. Gallic acid is the biosynthetic precursor of hydrolyzable tannins. This compound occurs extensively as complex sugar esters in gallotannins, such as penta-O-galloyl-glucose, but these are found only in a limited level in human diet. The correlated ellagitannins are also gallic-acid-derived tannins with a central core of an open-chain glucose and a characteristic carbon–carbon linkage between the glucose carbon-1 and the carbon-2 of the O-2 galloyl unit of the 2,3,5-nonahydroxyterphenoyl unit (NHTP group) (Figure 1). These are more common in human diet due to their presence in numerous fruits as well as nuts.

## 2. Tannins Organoleptic Taste Properties

Tannins contribute directly to major organoleptic properties, in particular to taste attributes such as astringency and bitter taste [11]. Several reports show that astringency is recurrently accompanied by bitterness, and that the perception of one could affect the perception of the other one, and this association is the source of confusion between these two taste properties [12,13,14]. It has been reported that when simultaneously perceived in the same product, astringency seems to be identified as a secondary attribute [12,13]. In fact, few works have studied the bitter/astringent properties of tannins, such as tannic acid fractions, procyanidins, and flavan-3-ol monomers, dimers, and trimers. In general, these studies reported that compounds with a lower molecular weight tend to be more bitter. In parallel, tannins with higher mDP, such as procyanidin oligomers, tend to be more astringent [15,16,17].

It is widely accepted that polyphenols, in general, can contribute to several health benefits. However, regarding tannins, when bitterness and astringency are perceived in high intensities, this can impair the consumption of a particular food product. Some studies invariably showed that taste is the key factor for food selection, overcoming nutritional or health value [17]. This creates a real obstacle to the food industry when designing (functional) health-promoting foodstuffs. Therefore, this motivates research on identifying which are the bitter and astringent compounds, understanding the food matrix effect on the perception of these taste properties of tannins, and to develop methods to modulate these taste properties while keeping the health-promoting compounds.

### 2.1. Bitter Taste

Bitter taste is crucial for wellbeing since it is triggered to avoid the intake of certain potentially harmful foodstuffs. This is important as many toxic plant metabolites taste bitter, thus, the corresponding taste receptor cells (TRCs) serve as warning sensors [18]. TRCs are localized in the oral cavity in groups of cells called taste buds, which are embedded in the epithelium of the gustatory papillae on the tongue and palate. Each taste bud can comprise from 50 to 100 TRCs [19]. These cells are characterized by the expression of members of the TASTE Receptor type 2 (TAS2R) gene family, which encode bitter taste receptors [20]. In humans, there are 25 taste receptors (hTAS2Rs) which are G protein-coupled receptors (GPCRs). Upon binding of bitter compounds to TAS2Rs, a bitter taste transduction response is triggered by the activation of Gαβγ protein subunits that subsequently activate several receptor potential channels and later lead to the increase of intracellular levels of Ca^2+^ and to cell membrane depolarization [21,22]. This taste transduction response is completed after the release of neurotransmitters, making it possible to recognize the intake of bitter compounds [23]. Bitter taste is usually assessed by sensory analysis [15,24,25]. Regarding tannins’ bitter taste, the few available data were mainly obtained by sensory analysis, and the list of studied tannins is very short.

There are a lot of important inconsistencies associated with these sensory assays [26,27]. Over the last two decades, more precise, reliable, and robust methods have used heterologous expression experiments of TAS2Rs to screen new agonists and/or to characterize the dose–response curve of the agonist–TAS2Rs pairs. The heterologous expression of TAS2Rs is a cell-based assay in which upon TAS2Rs activation by a certain compound, there is an increase of the intracellular Ca^2+^ (transduction pathway previously described), which can be determined by a fluorescent probe. Only in recent years has this approach been applied to tannin compounds. Therefore, concrete information about bitter taste elicited by tannins and which TAS2Rs are activated by tannins is still scarce. Sensorial analysis-based studies as well as TAS2Rs screening studies will be discussed further ahead.

Vidal and coworkers studied the taste and mouth-feel properties of tannins [28]. By sensorial analysis, they assessed that synthesized ethyl-bridged flavan-3-ols (Ethyl-B) with an mDP value of 5, resulting in a higher bitterness sensation compared to apple proanthocyanidin fractions with mDP values of 3 and 9 (Adp3 and Adp9). Several (astringent) sensory descriptors were evaluated in-mouth and after spitting (fine grain, medium grain, coarse grain, chalk, dryness, fullness, adhesive, pucker, overall astringency, and texture). Ethyl-B was rated intermediately between Adp3 and Adp9 for all the descriptors except for fine grain and bitterness (in which, ethyl-B rated higher). The authors suggested that the insertion of an ethyl-bond between each catechin unit contributed to the bitter taste.

Other sensorial analysis-based studies, such as the ones performed by Peleg and coworkers, have discovered that not only the molecular size of a tannin is crucial to its bitter taste, but also that stereochemistry and interflavanoid bonds play an important role in bitterness perception [15]. Overall, higher bitterness intensities were perceived in the presence of (epi)catechin monomers, then dimers (procyanidins B3, B4, and B6), and lastly in trimers (trimers C1 and C2). Within dimers, procyanidin B6 presented a significantly higher bitter sensation compared to procyanidins B3 and B4. Hufnagel and Hofmann reported that some of these procyanidins (dimers B1, B2, B3, and trimer C1) were perceived as bitter only at higher concentrations (0.4–0.5 mM) than astringent, perceived at lower concentrations (0.19–0.3 mM) [17].

Over the years, the work done by a few groups has given some insights into TAS2R activation by polyphenols. All these groups have used the previously described cell-based assay to identify which TAS2Rs were activated by a library of polyphenols belonging to different families [29,30,31,32,33,34,35].

Watanabe and coworkers studied the activation of TAS2Rs by tea catechins [31,32]. In their previous findings, a sensorial analysis has revealed that bitter taste was perceived in a higher grade for epicatechin gallate (ECG), followed by (−)-epigallocatechingallate (EGCG), epicatechin, and epigallocatechin (EGC) [36]. In accordance to the last statements, ECG induced stronger responses than EGCG to TAS2R39 in the following investigation, and ECG and EGC were similar to sensorial analysis. Furthermore, contrary to TAS2R39, hTAS2R14 appeared to be more strongly activated by EGCG [31]. The EC_50_ values obtained for hTAS2R14 activation by EGCG and ECG were 34 µM and 70 µM, respectively, while for TAS2R39, the values were 181.6 µM and 88.2 µM, respectively.

Soares and colleagues have found that pentagalloylglucose (PGG), a hydrolysable tannin, activated TAS2R5 and TAS2R39 with a very low EC_50_ (2.7 μM) [29]. In the same study, (−)-epicatechin activated three different TAS2Rs, TAS2R4, TAS2R5, and TAS2R39, whereas procyanidin trimer C2 activated TAS2R5. Tannins were identified as the first natural agonist for TAS2R5, displaying high potency. The catechol and/or galloyl groups appear to be important structural determinants that mediate the interaction of some of these tannins toward TAS2R5. Later, the same authors found that procyanidin B2-3-O-gallate also activated TAS2R5 and the ellagitannins punicalagin, castalagin, and vescalagin also activated TAS2R7 [30].

Table 1 presents a summary of the most relevant studies from the last two decades that explored the activation of hTAS2Rs by different tannins (condensed and hydrolyzable) and respective EC_50_. All these studies resorted to a similar methodology (as described in [37]), measuring the intracellular Ca^2+^ release (by Fluor-4-AM and/or Fluo-8-AM fluorescent probes) upon bitter agonist application.

Other groups have been exploring the inhibitory effect of some flavanones towards the activation of TAS2Rs by tannins, also monitoring the intracellular levels of Ca^2+^ ions. For example, Roland and coworkers tested the inhibitory behavior of fourteen flavanones towards the activation of hTAS2R39 in HEK-293 cells by (−)-epicatechingallate (ECG) [38]. These assays were conducted by two agonist/antagonist addition strategies: simultaneous and stepwise. In the first strategy, agonist and antagonist were applied at the same moment, whereas in the stepwise strategy, the antagonist was first applied. Only three flavanones have demonstrated inhibitory effects: 6-methoxyflavanone, 6,3′-dimethoxyflavanone, and 4′-fluoro-6-methoxyflavanone (order of increasing potency). It was observed that upon simultaneous addition, a maximal signal reduction of 65% was reached at 63 μM 4′-fluoro-6-methoxyflavanone, whereas a maximal signal reduction of 55% was reached at 500 μM of 6,3′-dimethoxyflavanone, and 6-methoxyflavanone showed negligible inhibitory activity. For the two first antagonists, further signal reduction could not be observed due to nonspecific signals of the compounds itself. 

For all flavanones the stepwise strategy was much more effective. The 4′-fluoro-6-methoxyflavanone antagonist achieved full inhibitor effects when applied prior to ECG (stepwise) with a half-maximal inhibitory concentration (IC_50_) of 102 µM. The inhibitory potency of these three antagonists has been assigned to substitutions on the B-rings. Additionally, apart from all the tested flavanones, 6-methoxyflavanone also showed an inhibitory effect towards the activation of hTAS2R14, but to a lower extent.

The same group has also been exploring the inhibitory effect of some proteins towards the activation of hTAS2Rs by tannins. This was followed by the simultaneous EGCG/protein addition strategy. Since β-casein, β-lactoglobulin, Ca-caseinate, Na-caseinate, gelatin B1, B2, B3, F1, and F2 binding affinities to EGCG were reported earlier [39], these complexing agents were tested for their potential bitter taste-masking effects [40]. An EC_50_ of 161 µM for EGCG was reported for hTAS2R39 activation. In most cases, the complexation of EGCG with these proteins resulted in a decrease of hTAS2R39 activation in cellular assays. Particularly, the EGCG–protein complexes using β-casein, gelatin F1, and Na-caseinate, resulted in bitterness reduction of 93%, 46%, and 34%, respectively. To complement these findings, a sensorial analysis contemplating a trained sensory panel of 13 persons was performed. The results showed that sensory analysis was in accordance to the in vitro prediction/screening of the potential of food proteins for bitter masking.

### 2.2. Astringency

Astringency is defined as dryness, tightening, and puckering sensations perceived in the oral cavity during the intake of astringent compounds. A common sensory example used to explain this sensation to a nonscientific audience is the sensation experienced during the intake of unripe bananas or persimmons. 

The term astringency, with its origin in latin *adstringere* meaning “to bind”, can be induced by the binding of alums, acids, alcohols, and tannins. In fact, the term tannin is used to define a particular group of polyphenols that has the special ability to bind to proteins. The origin of this term dates back to tanning practice, the treatment to transform animals’ skin into leather. This was due to the interaction between the polyphenols present in the plant-based extracts used with the skin proteins, collagen in particular. Since that time, the data gathered by the scientific community has shown that the interaction between tannins and proteins has many more implications besides that first ancestral application. Depending on the tannin and protein targets, these interactions have important outcomes related to the health, structural functionality, and organoleptic properties of food products. In fact, in food, tannins and some other related polyphenols are the major compounds responsible for astringency.

**Figure 2:**
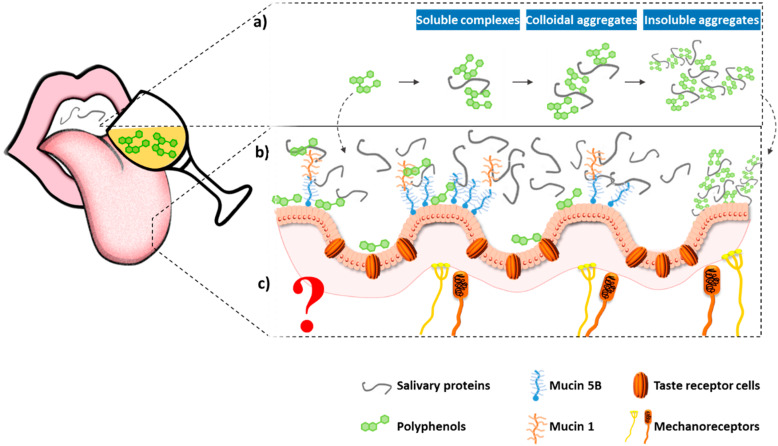
Proposed mechanisms for astringency onset: (**a**) interaction and precipitation of salivary proteins (Topic 2.2.1); (**b**) interaction of phenolic compounds (PC) with oral cells and/or mucosal pellicle (Topic 2.2.2); (**c**) activation of oral mechanoreceptors. For details on the mechanisms see text.

Despite the longstanding disagreements on the molecular basis of astringency, in recent years, a considerable extension of the knowledge on this topic has been observed. In 1954, Bate-Smith proposed the first mechanism to explain molecular astringency perception, assuming that it was due to the interaction and precipitation of (salivary) proteins (Figure 2a) [41,42,43]. According to Bate-Smith, the primary reaction whereby astringency develops in the palate is by precipitation of glycoproteins in the mucous secretions of salivary glands [43,44]. Precipitation of glycoproteins/mucoproteins takes them out of saliva, causing a less viscous and consequently less lubricating fluid. Moreover, the precipitated complexes are “free” to adhere to the mucosa where they can form a sticky residue, which should certainly raise the coefficient of friction among mucosal surfaces. Along the years, evidence has been accumulated, suggesting that a higher interaction between tannins and particular (salivary) proteins, for example, proteins that are flexible “open” proteins and those rich in the amino acid proline, such as the proline-rich proteins (PRPs). This was first observed by Hagerman and Butler who compared the interaction of different polyphenols to a series of “open” vs. compact globular proteins [4,5,6,7,8,9,10,11,12,13,14,15,16,17,18,19,20,21,22,23,24,25,26,27,28,29,30,31,32,33,34,35,36,37,38,39,40,41,42,43,44,45,46,47]. The linking between PRPs and tannins was further supported by subsequent studies with rats, which showed that PRPs were induced by isoproterenol and condensed tannin diets [47,48,49,50]. In rats and mice, PRPs were virtually absent until they were induced by dietary tannins; in humans and ruminants, these proteins are constitutive and exist in amounts that appear to reflect the predictable level of tannins in their usual diets. Since that time, it has been hypothesized that PRPs, in particular basic PRPs, could be a first line of defense against the detrimental effects of tannins in the diet [49,51].

#### 2.2.1. Interaction between Tannins and Proteins

Since the first findings that PRPs have stronger interaction with tannins, a large number of studies have focused on the physicochemical characterization of these interactions. These interactions between tannins and proteins have been the focus of several studies that tried to understand the effect of tannin and protein structure, solvent (e.g., ethanol), temperature, other compounds (e.g., polysaccharides), pH, and ionic strength.

Salivary PRPs have a characteristically high proline content, which accounts for 25% to 42% of the amino acids in PRPs. These proteins are classically divided into basic, glycosylated, and acidic PRPs based on specific differences in their amino acid content [52]. Basic PRPs are rich in basic amino acids and have high isoelectric points (pI > 10), glycosylated PRPs are derived from basic PRPs, but have carbohydrates linked to some amino acids, and acidic PRPs are rich in acidic amino acids, namely glutamate.

Regarding tannins’ structure, some key features are the molecular weight, polymerization degree, galloylation degree, stereochemistry, and conformation, such as the interflavanic linkage. A universal rule is difficult to achieve because this interaction is highly dependent on the tannin–protein pair, but some general trends can be identified: greater interaction occurs between tannins with a higher polymerization degree, molecular weight, and number of galloyl groups (galloylation degree) [53].

Bacon and colleagues found a relationship between the level of tannin galloylation (a series of flavan-3-ol monomers from tea and a commercial tannic acid) and the affinity of binding to parotid salivary protein [54]. The same author investigated the interaction of a series of hydrolyzable tannins with the major classes of PRP (acidic, basic, and glycosylated) and observed a structure–binding relationship with the levels of tannin galloylation, hexahydroxydiphenoyl esterification, and degree of polymerization [55]. They also observed that all the classes of PRP appear to have no exclusive affinity for a particular type of hydrolyzable tannin. De Freitas and Mateus also observed increased interaction with proteins (PRPs, amylase, and BSA) due to galloylation, since the procyanidin dimer B2-3′’-O-gallate had greater interaction than its counterpart, procyanidin dimer B2 [56,57]. These authors also observed the effect of the polymerization degree (higher interaction for procyanidin trimer C1 than for procyanidin dimers or monomers), the effect of stereochemistry of flavan-3-ols ((+)-catechin had a higher interaction for PRPs than (−)-epicatechin), and the effect of interflavanic bonds (procyanidin dimers C(4)–C(8) had consistently greater interaction than their counterparts C(4)–C(6)). Charlton and colleagues also showed that a higher degree of galloylation (from EGCG, trigalloylglucose, tetragalloylglucose, and pentagalloyglucose with two, three, four, and five galloyl groups, respectively) led to a higher extent of interaction [58]. Numerous works have reported a similar trend [59,60,61,62,63,64,65,66,67].

The position of the galloyl group is of critical importance. For instance, the interaction of 2,3,6-trigalloylglucose has been reported to have a higher interaction than the 2,3,4-trigalloylglucose structure [64]. Poncet-Legrand observed aggregation between epicatechin gallate and a poly(l-proline) model peptide while epigallocatechin did not [68]. The position of the galloyl group seemed to be critical for these interactions.

Recently, it has been shown that the perception of different astringent subqualities and descriptors can be related to the structure of polyphenols (tannins and non-tannins). Zhuang and colleagues found that the condensation of flavan-3-ols and hydroxylation degree in the B-ring of flavonoids contribute to the “coarse” astringent taste, while the acylation of polyphenols had a major contribution to the “grassy” astringent taste [69]. The results indicated that the acylation of phenolic acid may increase the astringency intensity, as well as the galloylation and polymerization of flavan-3-ols and 7-O-glucosylation of kaempferol.

Regarding the protein structure effect, a general trend is that random coiled and more “open” proteins have a higher interaction with tannins. This is a common structural trait for proteins that are rich in proline. PRPs are not common among biological proteins [70]. The presence of proline residues in proteins implies some structural constraints, since it is the only amino acid where the side chain connects to the protein backbone twice, forming a pyrrolidine side chain. This feature significantly restricts the phi (φ) angular range in peptide bond formation, giving exceptional conformation rigidity and therefore affecting the secondary structure of proteins [70]. In fact, proline residues tend to be excluded from alpha helices and beta sheets. Therefore, PRPs face tremendous constraints, since some of these residues account for 50% of the protein sequence. The comparison between the interactions of PRPs or derived peptides and other globular proteins toward tannins has been the focus of several works [71]. In general, a higher interaction with PRPs or derived peptides has been reported. There are inconsistencies of the data when comparing the reactivity of the different families of PRPs. Lu and colleagues observed that basic PRPs (bPRPs) were very effective in establishing insoluble complexes with both condensed tannin and tannic acid, while nearly no tannin bound to acidic PRPs (aPRPs) and glycosylated PRPs (gPRPs) [72]. Sarni-Machado and colleagues found that gPRP–(condensed)tannin interactions led to complexes that remained soluble, whereas those arising from nonglycosylated PRP–tannin interactions were precipitated [60]. On the other hand, Soares and colleagues found that aPRPs were the ones with the highest interaction toward condensed and hydrolyzable tannins, using both in vitro and in vivo approaches [73,74,75].

Altogether, considering tannin and protein structure, it seems important to have a complementarity between the polydentate ligand (tannin) and the protein that is maximized by conformational flexibility in both components.

Regarding the mechanism of interaction, it has been proposed that the interaction between PRP and tannins could occur by a three-stage mechanism [76]. In the first stage, the saturation of the interaction sites occurs, followed by protein aggregation into colloidal complexes at higher tannin/protein ratios (second stage). Further increase of this ratio lead to haze formation (third stage).

Another important physicochemical feature of tannin–protein interaction is the type of noncovalent interactions involved on the complexes formation. It is commonly observed that these interactions occur mainly and firstly through a hydrophobic stacking between a tannin galloyl ring and the pyrrolidine ring face of proline residues. Then, secondary hydrogen-bonding effects help to stabilize the interaction. This was observed by Murray and colleagues for the interaction between two synthetic peptides rich in proline residues with PGG [77]. However, the first report of the importance of hydrophobic effect on these interactions goes back a long time ago, in 1980, where Oh and colleagues performed several experiments with immobilized condensed tannins on a Sepharose column and followed the adsorption of protein [78]. Baxter and colleagues found the same conclusion for the association of a synthetic 19-residue PRP fragment with a series of polyphenols. Additionally, these authors found that, in a sequence of proline residues, the first proline is a favored binding site. The predominance of hydrophobic stacking on these interactions is widely reported [58,79,80,81,82].

Regarding the effect of temperature, solvent, and pH, there is a higher variability and less data available and the effects seem to be more dependent on the protein–tannin pair. The first to suggest that pH could affect protein–tannin interaction were Hagerman and Butler [83]. It was pointed that the lowest solubility of polyphenol–protein aggregates (the highest interaction) occurs at 0.3–3.1 pH units lower than the isoelectric point of the proteins [84]. In fact, Naczk and colleagues found a higher interaction between pH 4 and 5, and lower interaction at pH values lower (3.5) and higher (6.0) than these ones, for the interaction between BSA and polyphenols from blueberry leafs [85]. The same was observed for the interaction between mucin and several procyanidins (dimer B4, tetramer, and oligomeric fractions) [86]. The interaction was significantly higher at pH 5.0 and almost completely prevented at pH 2.6. Kawamoto and Nakatsubo that studied the interaction between BSA and tetragalloyglucose over a range of pH values (3 to 7) and found that the interaction was higher at pH 4 [64]. On the other hand, Charlton and colleagues found no effect of pH (between pH 3.8 and 6.0) on the interaction between EGCG, ECG, trigalloyglucose, and tetragalloyglucose with PRP peptides. These last authors observed a decrease in the affinities with temperature increase. On the other hand, several years ago, Oh and colleagues found that complex formation between condensed tannins and gelatin or poly-L-proline increased with increasing temperature, as expected for hydrophobic interactions [78]. Hofmann and colleagues found that the interaction between hydrolyzable tannins (PGG, castalagin, and grandinin) toward BSA increased along with temperature [71]. Despite this general trend, the extension of the temperature effect was quite different for the different compounds, with grandinin being most affected by temperature.

Regarding the solvent effect, most works focused on the effect of ethanol, since tannins occur in alcoholic beverages, but some works also focused on the effect of dimethylsulfoxide (DMSO). In general, the presence of ethanol (low percentages between 4% and 12%) decrease the interaction between tannins and proteins [87]. Pascal and colleagues found that the aggregation of a gPRP (II-I) to EGCG was disrupted by the addition of 12% ethanol [59]. Brandão and coworkers observed that ethanol and DMSO can disrupt the interactions between mucin and several procyanidins (dimer B4, a tetramer and oligomeric fraction) [86]. A different trend was observed by Obreque-Slíer and colleagues [88]. The authors observed that enological concentrations of ethanol (13%) exacerbate astringency and salivary protein−tannin interactions and precipitation of both proanthocyanidins and gallotannins.

A recent work has also hypothesized another aspect of the protein–tannin interaction that has never been considered before and could be related to astringency, namely the time required to dissociate the formed complex [89]. This observation was made by studying the interaction between mucin (immobilized on a C1 chip) and three oenological (ellagitannins, gallotannins, and proanthocyanidins) tannin extracts by surface plasmon resonance (SPR). Typically, in all SPR sensorgrams, two steps are clearly distinguished, the association step between the different tannins and immobilized mucin, and the dissociation step that starts when the tannin injection is stopped and only the buffer is flowing. These sensorgrams allow one to determine the kinetic and thermodynamic constants of the interactions. It was found that the kinetic association constants (ka) of the three types of tannins is much greater than their corresponding kinetic dissociation constants (kd). These data indicate that there is a strong interaction between mucin and tannins in which the association curve increment is very fast and the values of the dissociation curves are maintained for a long period of time. These behaviors suggest that the astringency of these tannins will be felt quickly and that it will persist for a long time. Furthermore, ellagitannins have a ka 14.8 times higher than gallotannins and 22.8 times higher than seed tannins, which would suggest that ellagitannin may initiate astringency perception faster than the other tannins. In contrast, the three tannin types have similar kd values.

#### 2.2.2. Other Mechanisms for Astringency

For quite a long time, protein–tannin interaction/precipitation has been considered the central mechanism of astringency perception. However, this phenomenon does not explain all the described sensations, and some inconsistencies between in vitro (interactions) experiments and sensory studies have been reported. For example, low-molecular weight polyphenols were found to elicit astringency by complexing salivary proteins without precipitation [90]; some polyphenols (not tannins) that do not precipitate salivary protein were reported as highly astringent with thresholds 16,000 times lower the threshold determined for EGCG [91]. In this way, Schwarz and Hofmann proposed that the quantity of the nonbound, “free” astringent stimulus in the saliva might be more closely related to the sensory perception of astringency than the amount complexed/precipitated by salivary proteins [92]. This was also supported by the data collected by Nayak and colleagues [93]. These authors explained their observations, proposing that salivary proteins in solution inhibit the sensation of astringency.

It has been also proposed that astringency is a tactile sensation caused by increased friction due to decreases in salivary lubrication between oral membranes [94]. Recently, a strong correlation between the oral friction coefficient and the sensory intensity perception of astringency has been reported [95]. Prinz and coworkers showed that tannic acid, a model commercial mixture of hydrolyzable tannins, significantly reduces the lubricating qualities of human saliva by both decreasing its viscosity and increasing friction, both factors lending support to the notion that astringency is a tactile phenomenon [96]. On the other hand, Laguna and colleagues found a positive correlation between viscosity and astringency [97]. These authors studied the density and rheological properties of a mixture between six different model-wine formulations containing one or multiple wine components (ethanol, mannoproteins, glycerol, and tannins) with human saliva. They found that the viscosity of samples with tannins was the highest due to the formation of complexes between the model-wine and salivary proteins.

Other parameters besides salivary proteins were found to affect the perception of astringency, such as saliva flow rate. Low saliva flow subjects were found to have higher intensity of astringency, longer time to reach maximum intensity, and a longer duration time [98]. A similar trend was observed by Condelli and colleagues, who observed that astringency intensity perception proved to be inversely related to saliva flow rate and haze developing capacity [99]. Moreover, these authors found no significant correlations between saliva protein concentration and intensity of astringency perception.

In 1993, the hypothesis that the epithelium itself could contribute to astringency was raised. Green and colleagues suggested that the astringent molecules could interact with proteins in the epithelium itself and these interactions could change the physical characteristics of the mucosa, which is expected to have complex effects on the mechanical sensitivity to movement [13]. Recent data suggest that oral epithelial cells can bind to tannins (Figure 2b). The first report was made in 2009 by Payne and colleagues [100]. After this report, others followed with consistent data [101,102]. In fact, oral cells can contribute to this process in two distinct ways: as the main components of the epithelium of the oral mucosa, or as the basis for the formation of the mucosal pellicle, a thin layer of salivary proteins adherent to the epithelial oral cells. The importance of the mucosal pellicle to astringency was already proposed by Nayak and colleagues [93]. However, recently, Ployon and colleagues developed an elegant method to study the impact of two tannins (EgC and EGCG) on the mucosal pellicle structure and properties [103]. Aggregates with the proteins at the mucosal pellicle (mucin 5B) were observed above the sensory detection threshold (0.5 and 1 mM) and their size increased with tannin concentration and with galloylation. In addition, 3 mM EgCG resulted in higher friction forces. A new cell-based method was also developed by Soares and colleagues to study the contribution of the different oral constituents (oral cells, mucosa pellicle, and salivary proteins) to the tannin-binding process [104]. These authors studied the interaction of a mixture of oligomeric procyanidins, containing all the procyanidin dimers and some procyanidin trimers and galloylated dimers. This more complex model revealed a higher interaction (synergism) for the model with all the referred oral constituents when compared to the interaction with individual constituents, the procyanidins interaction only with oral cells, or only with salivary proteins. Regarding procyanidin structure, a significant interaction was observed for the procyanidin monomer ECG, procyanidin dimers B7 and B2g, and procyanidin trimer C1.

The possibility that mechanoreceptors could be involved in astringency had been also raised many years ago (Figure 2c) [13,105]. Mechanoreceptors are neurons classified according to their size and their receptive fields into type I and type II [106]. Additionally, they are further classified into rapidly adapting (RA) or slowly adapting (SA) receptors. The mechanoreceptors of the oral cavity include: Ruffini endings, Merkel cells, Meissner cells (lamellated corpuscles), and free nerve endings [107]. While oral mechanoreceptors seem to function like those of the skin, they show smaller receptive fields and lower activation thresholds [106]. In fact, most of the knowledge about oral mechanoreceptors is by analogy to skin mechanoreceptors. Given that both the lingual nerve and the chorda tympani nerve contain RA mechanoreceptors that respond to light stroking of the tongue [108], it is likely that depriving the mucosa of its natural lubricant changes the neural inflow from these fibers. A recent paper brought new light into this subject; an elegant study by Schöbel and colleagues focused on astringency perception in human subjects, showing that the trigeminal nerve is involved in astringency perception [109]. Additionally, these authors screened primary mouse trigeminal ganglion neurons for activation by different polyphenol (monomers and polymeric red wine) astringent compounds and observed activation by the compounds with one or several galloyl moieties, whereas substances lacking the moiety did not stimulate or only stimulated weak responses. However, concrete data about which mechanoreceptors directly lining the oral cavity are activated is so far unknown.

## 3. Tannin–Macromolecule Interactions: Effects on Organoleptic Properties

During food and beverage processing, tannins can interact with different macromolecules, such as proteins and polysaccharides, by the mechanisms described above. Although tannins are always plant-derived compounds, depending on the food matrix, other macromolecules can be also derived from plant or from animal sources. Various physicochemical phenomena can occur due to these interactions that have an impact on the nutritional, technological, and organoleptic properties of food.

### 3.1. Tannin-Protein Interactions: Beyond Astringency

Tannins from foodstuffs can interact with proteins in very different stages of the food chain. The interactions presented so far occur in the first contact with the human body, the oral cavity. After this, some tannins can reach the gastrointestinal system and interact with other (biological and nonbiological) proteins, such as gastric enzymes or other food proteins, among others. The interactions with other biological proteins were reviewed previously and will not be further reviewed here [110]. Tannins may interact with food proteins in different stages of the food chain, either in the plant or during food processing, and these interactions can affect several the sensory properties of tannins as well as their bioavailability, certain technofunctional properties of food proteins, and their digestibility.

The occurrence of haze or bottle precipitation are a common problem in some plant-based beverages, particularly apple-derived beverages (e.g., fruit juice, ciders), beer, and wine. This visual attribute is particularly important because it is perceived before taste and can lead to product rejection by consumers. Haze can occur by numerous causes, but one of the most common is the interaction of polyphenols and proteins, leading to the formation of aggregates [111]. The main polyphenols identified in permanent haze are condensed tannins, in particular proanthocyanidins and propelargonidins [112], while other polyphenols are involved in cold haze formation. In fact, proanthocyanidin dimers are thought to play the most significant role in beer haze, while trimers, tetramers, and higher proanthocyanidin oligomers are less likely to survive the brewing process [113].

Interestingly, some plant-based PRPs have been described as the most haze-active proteins [111]. Hordein, the barley prolamin with around 20% proline residues, is known to be the main beer-haze active protein [111]. Some PRPs have been found in apple juice [114] and grape seeds [115].

In order to avoid or delay haze formation, several fining and clarification processes are widely used in the wine and fruit juice industries [116]. The key mechanism behind the fining and clarification processes is tannin–protein interaction. These processes are mainly used to stabilize the final beverage by removal of highly reactive tannins, but they are also applied to modulate the final taste properties [117,118,119], namely astringency and bitterness.

In this process, several proteins are commonly used, but the most common are casein, gelatin, and egg albumin. There are some reports showing that the use of these fining agents, also lead to a decrease in the level of astringency and bitterness in the press fraction of wines, particularly in red wines [120]. Gelatin decreases astringency in red wines by dropping tannin levels and tends to remove more higher molecular weight galloylated proanthocyanidins than lower molecular weight tannins [121]. A casein fining solution is used in particular for the treatment of astringency and for the clarification of white and rosé wines, but is also sometimes used with red wines. These food proteins have been also studied for their potential as bitter-masking proteins in TAS2R39 activation by EGCG [40]. The potential bitter-taste-masking effect of β-casein, β-lactoglobulin, Ca-caseinate, Na-caseinate, and different types of gelatin (B1, B2, B3, F1, and F2) were studied since these complexing agents were previously known to have binding affinities to EGCG [39]. In most cases, the complexation of EGCG with these proteins resulted in a decrease of hTAS2R39 activation in cellular assays. Particularly, EGCG–protein complexes using β-casein, gelatin F1, and Na-caseinate resulted in a bitterness reduction of 93%, 46%, and 34%, respectively. To complement these findings, a sensory analysis contemplating a trained sensory panel of 13 persons was performed. The results showed that sensory analysis was in accordance with the in vitro prediction/screening of the potential of food proteins for bitter masking.

There is an increasing interest in plant-based proteins to be used as alternative fining agents to animal protein. A wide variety of commercial preparations of plant-based proteins from soy, gluten wheat, rice, potato, lupin, or maize had been proposed for oenological use with the name of vegetable proteins. A recent review was published on this topic [122]. Some of these proteins were reported to precipitate galloylated (condensed) tannins depending on their origin and their molecular weight [123]. For instance, the treatment of wine with zein proteins extracted from corn gluten were able to decrease wine turbidity due to removal of phenolic compounds, such as anthocyanins and proanthocyanidins, but, at the same time, it did not significantly affect red wine color and “bouquet” [124].

In addition to haze and taste properties, tannin–(food) protein interactions can affect other sensory properties of food and, in fact, they can be used for improvement of food quality. For example, the conjugation of proteins and nonpolar tannins can raise the surface hydrophobicity of the modified proteins, and thus enhance the emulsifying properties of the native proteins [125].

Protein–EGCG conjugates have been reported to exhibit better antioxidant activity than unmodified proteins. The interaction between gelatin with gallic acid and rutin have shown increased gel strength, thermal stability, and decrease in swelling. The interaction of bovine β-lactoglobulin with (−)-epigallocatechin contributes to the functionality of dairy products. An extensive description of these interactions can be found elsewhere [125].

### 3.2. Tannin–Polysaccharide Interaction: Modulation of Taste Properties

Food product selection is greatly dependent on the product’s sensory properties and on the intensity of the sensations experienced by the consumers [126]. The previously described tannin taste properties, bitterness and astringency, are usually referred to as nonpleasant, eliciting negative consumer reactions when perceived at high intensities [127]. However, in some products such as red wines and beer, a balanced level of astringency is desirable for their quality. Polysaccharides are widely used in the food industry as food additives (thickeners, emulsifiers, gelling, and texturizing agents) affecting the sensorial properties of products and therefore consumer choice [128]. They have been associated with mouthfeel, and modify the sensory properties of products, especially of wine [129].

#### 3.2.1. Polysaccharide Effects on Astringency

The first evidence on the effect of polysaccharides on astringency perception and subsequently on protein–tannin interactions was proposed by Ozawa and colleagues, explaining the phenomenon during fruit ripening [130]. Those authors observed that pectins, the main constituents of cell walls, suffer enzyme-catalyzed depolymerization and de-esterification during ripening, and the large insoluble molecules present before ripening were degraded, resulting in small soluble molecules. These soluble polysaccharides compete with salivary proteins for polyphenolic substrates, which leads to a modification in astringency perception and response [130]. The information available in the literature about ternary (salivary) protein–tannin–polysaccharide systems is not as well documented as the ones regarding binary tannin–polysaccharide systems. This is probably due to technical limitations of structurally characterizing these kinds of complexes. However, in recent years, several studies have reported on the effect of polysaccharides on the interaction between (salivary) proteins and tannins (ternary system) and on astringency perception. These studies will be discussed below.

**Figure 3:**
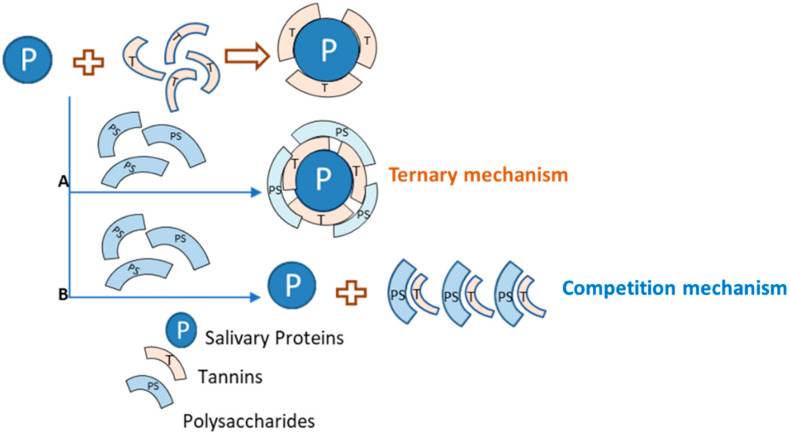
Possible mechanisms ((A): ternary mechanism and (B): competition mechanism) involved on the inhibition of the aggregation of tannins and salivary proteins by polysaccharides. P: salivary proteins, T: tannin, PS: polysaccharide. Adapted from [131].

According to the literature [131,132,133], polysaccharides can disrupt salivary protein–tannin interactions by different mechanisms: by a competition mechanism in which polysaccharides compete with salivary proteins by tannin binding; or by the formation of a ternary protein–tannin–polysaccharide complex, enhancing its solubility in aqueous medium (Figure 3).

These two mechanisms have been supported by several research works. The first data concerning ternary systems was obtained using model polysaccharides (e.g., pectin, cyclodextrin, arabic gum, xanthan) and model proteins (e.g., BSA, α-amylase). For instance, Gaffney and coworkers studied the association of polyphenols (methylgallate and trigalloylglucose) in aqueous media with caffeine (shares some similarities with peptides) and with α- and β-cyclodextrin in binary and ternary systems using NMR spectroscopy and microcalorimetry [134]. The results showed that the extent of association of polyphenols with substrates such as proteins and caffeine is likely to be affected by the presence of polysaccharides that could develop a secondary structure containing hydrophobic cavities in aqueous media. These hydrophobic cavities of polysaccharides may encapsulate the aromatic groups of polyphenols, inhibiting the polyphenol’s binding ability and supporting the hypothesis that cyclodextrin acts via a competition mechanism. Other authors also observed that the hydrophobic cavity of cyclodextrins is able to incorporate different tannin subunits, catechin and epicatechin [135], as well as other polyphenols such as chlorogenic acids [136], quercetin [137], and anthocyanins [138], among others. Pectin was used as a model polysaccharide by Luck et al., who showed that this polysaccharide was able to inhibit the precipitation of proline-rich gelatin and sodium caseinate by PGG [132].

Similar to the interaction between proteins and tannins, it seems that main driving forces responsible for the interaction between tannins and polysaccharides in solution involve hydrogen bonds and hydrophobic interactions [139,140,141,142]. The structural features of both polysaccharide and tannins are also of relevant importance, since they must have a suitable size and flexibility to interact readily [131,142,143,144]. Furthermore, the ionic character of polysaccharides, which is related with the polarity of these macromolecules, may influence polysaccharide–tannin interactions. For instance, de Freitas and colleagues have evaluated the effect of several polysaccharides—ionic and neutral—on the interaction between grape seed procyanidins and BSA [140]. The authors noted that ionic polysaccharides, such as pectin, xanthan, polygalacturonic acid, and arabic gum, were more efficient in reducing the interaction between BSA and procyanidins than the neutral polysaccharides, such as β-cyclodextrin, arabinogalactan, dextran, and glucose. The same authors studied the influence of the tannin structure on the disruption effect of polysaccharides (e.g., xanthan, arabic gum, and pectin) on protein–tannin aggregates [131]. They observed that all polysaccharides were more effective with low mDP procyanidins and the effectiveness of polysaccharides to disrupt BSA–tannin aggregates increased with the polarity of polysaccharides in the order xanthan > pectin > arabic gum. Furthermore, they noted that xanthan was efficiently able to encapsulate low polymerized tannins in its hydrophobic pockets via a competition mechanism (similar to the hydrophobic cavities of cyclodextrin). Unlike xanthan, pectin was not able to encapsulate tannins, seeming to form soluble ternary complex with BSA–tannin or only with tannins, thus competing with BSA for tannin binding [131]. Carvalho and colleagues also noted that xanthan’s ability to inhibit BSA–tannin complex formation was higher for condensed tannin fractions of smaller size and lower complexity [144]. The influence of the polysaccharide structure as well as the mDP of the tannin fraction used on the driving mechanism, competitive or associative, by which protein–tannin aggregation is inhibited was also assessed by Soares and coworkers [145].

More recently, the studies have been developed using salivary proteins or peptides isolated from human saliva instead of model proteins. For instance, in the work developed by Soares et al., the inhibitory effect of some polysaccharides—pectin, arabic gum, and polygalacturonic acid—on the interaction between salivary proteins and condensed tannins (grape seed procyanidins) was studied by HPLC and SDS-PAGE [146]. The results showed evidences that pectin was the most efficient in inhibiting salivary protein–tannin precipitation, followed by arabic gum and polygalacturonic acid. Moreover, pectin and polygalacturonic acid seemed to favor the formation of a ternary salivary protein–tannin–polysaccharide complex, while arabic gum competes with proteins for tannin binding (competition mechanism).

Wine polysaccharides have been also tested in different approaches to study astringency perception. In addition to tannins, polysaccharides are one of the most important macromolecules found in wine and have been widely isolated and characterized during the past decade [147,148,149]. They can be grouped into three major families: (i) polysaccharides rich in arabinose and galactose (PRAGs), which include arabinans, arabinogalactans, and arabinogalactan-proteins (AGPs), (ii) those rich in rhamnogalacturonans (RG I and RG II), which come from the pectocellulosic cell walls of grape berries, and (iii) mannoproteins (MPs), another group of wine macromolecules, produced by yeasts (*S. cerevisae*) during fermentation and during the aging of wines. Vidal and colleagues reported that red wine was composed of 42% AGPs, 35% MPs, 19% RG II, and 4% RG I [148]. AGPs and MPs are usually associated with neutral polysaccharides while RG II has a more acidic character. It has been described that these polysaccharides may influence the perceived astringency of wines, which is related to their structure and physicochemical properties (e.g., ionic character and viscosity). It was reported that RG II was able to significantly decrease the attribute ratings associated with the astringency of the model wine in the absence and presence of procyanidins, whereas the neutral wine polysaccharide fraction containing AGPs and MPs had less effect on reducing the ratings for these attributes. Indeed, RG II significantly affected the attributes related to the astringent sensations felt on the mouth surfaces (roughness and dryness), which are possibly caused by changes in mouth lubrification, and it is likely that the viscosity of RG II compensates the decrease of lubrication. Furthermore, acidic polysaccharides, such as RG II, are more able to reduce astringency than the neutral ones, specifically its coarse, chalk, and pucker subqualities, possibly due to a higher affinity for interaction with some wine compounds, such as tannins, which are responsible for these unpleasant subqualities [129,150].

However, different behavior was observed in the work by Carvalho et al. [151]. These authors studied the effect of these polysaccharides on the interaction between grape seed tannins and salivary proteins (α-amylase and IB8c, a PRP) by light scattering. They noted that the most acidic fractions of AGPs have the ability to inhibit the formation of aggregates between grape seed tannins and the two different salivary proteins. On the other hand, RG II has the same ability toward α-amylase, but not IB8c under the conditions of the study. This study showed the importance of protein structure in the polysaccharide’s effect, as well as the polysaccharide’s ionic character. Furthermore, these polysaccharides were tested at concentrations in which they are present in wine, which could mean a real influence in wine astringency [151]. The effect of wine polysaccharides on astringency perception was also evaluated by Quijada-Morín and colleagues [152]. The results provided evidence that RG II and MPs were able to “smooth” wine astringency perception, probably due to their ability to interact with wine proanthocyanidins, contributing to a lower amount of these compounds available to interact with salivary proteins [152]. In addition to sensory evaluation of the effect of polysaccharides on astringency perception, some molecular approaches have been developed to understand how it occurs at a molecular level. For instance, Brandão et al. performed several studies on the effect of different polysaccharides on the interaction between tannins and salivary proteins [153,154,155]. They tried to understand the underlying molecular mechanisms of the inhibitory effect of several polysaccharides (from wine and white grape skins) on the interaction between tannins (condensed and hydrolyzable) and salivary proteins. In all of the different molecular approaches, the authors tried to simulate what happens during ingestion, where polysaccharides first interact with tannins, and only when they come into contact with salivary proteins. In general, they observed that polysaccharides can act by a competition or ternary mechanism, depending on several structural features of the macromolecules involved. The results also suggested that polysaccharides’ effects are affected by the differences of polarity of the molecules, which influences the main driving forces of those interactions (hydrogen bonds and hydrophobic interactions) [153]. In these works, the authors also verified that the presence of salts (e.g., NaCl) can decrease the inhibitory effect of polysaccharides [154]. Another interesting finding is that the interactions and consequently the effect of polysaccharides is significantly affected by the presence of the molecules alone or in a mixture. For instance, isolated polysaccharides seemed to be less effective in inhibiting protein–tannin interactions when salivary proteins are present together in saliva in comparison with the proteins individually [153,154]. On the other hand, a pectic polysaccharide fraction is able to inhibit salivary protein–tannin interactions even when proteins are present together in saliva [155].

Another research on the molecular mechanisms by which polysaccharides can affect protein–tannin interactions was developed by Manjón and coworkers [156]. These authors studied the molecular mechanisms by which commercial MPs could modulate astringency elicited by tannins through ITC and molecular dynamics simulation. The results obtained indicate that MPs could affect astringency by the formation of ternary aggregates with different solubilities or by preventing flavanol–PRPs interactions by a competitive mechanism. This MPs effect is different among the several MPs used, depending on the size and the compositional characteristic of the MP fraction [156]. The effect of wine polysaccharides on the interaction between wine flavan-3-ols and BSA was also studied at the molecular level by another research approach using fluorescence [63]. The results of the previous work indicated that RG II and MPs were the major polysaccharides to significantly modify the interaction of flavan-3-ols with BSA. Watrelot and colleagues also studied the influence of red wine polysaccharides on the interaction between tannins and proteins (BSA) or a hydrophobic surface [157]. The authors noted that RG II, the proportion of pigmented tannins, and the mDP seemed to favor protein precipitation either through the formation of insoluble tannin-polysaccharide aggregates or because of protein–polysaccharide interactions.

In a wine context, the interactions between polysaccharides and tannins are expected to occur during winemaking. The formation of tannin–polysaccharide complexes may influence their association with salivary proteins, which then leads to a change in astringency perception. These tannin–polysaccharide interactions are dependent on the structure and conformation of the condensed tannins (higher mDP will increase the interaction) as well as the structure and composition of the polysaccharides. For instance, the most neutral fraction of AGP (lower uronic acid content) has no impact on wine tannin aggregation, but this self-aggregation was greatly inhibited by the most acidic AGP fraction (higher uronic acid content) [158,159]. On the other hand, MPs were able to inhibit tannin aggregation, therefore preventing haze-formation and precipitation during wine ageing [158]. This effect was mainly observed for low molecular weight MPs [160]. Concerning RG II, it was shown that RG II monomers had no impact on tannin aggregation, while the dimers (monomers cross-linked with a borate diester linkage) may increase aggregation [158].

Table 2 presents a summary of the main relevant studies on the interaction of different polysaccharides and tannins (binary system) as well as the effect of polysaccharides on the interaction between (salivary) proteins and tannins (ternary system).

#### 3.2.2. Effect of Polysaccharides on Bitterness

In comparison with astringency, there are only few studies in the literature concerning the effect of polysaccharides on bitterness. Furthermore, most of the studies reported are based on sensory analysis, which is not a very robust technique. For instance, Smith and colleagues evaluated the effect on bitter taste of the addition of carboxymethylcellulose (CMC) to a grape seed tannin solution [161]. The authors noted that the maximum intensity and total duration of bitterness were not significant affected by increasing the viscosity; however, the onset of bitterness was significantly delayed. Different evidences were observed by Troszyńska and coworkers [162]. They investigated the influence of food gums (guar, xanthan, and arabic gum) and CMC on bitterness caused by caffeine. The authors observed that only CMC was able to suppress the bitter taste of caffeine. Calvino and colleagues also observed that the intensity in perceived bitterness elicited by caffeine could also be suppressed upon the addition of sucrose [163].

Regarding wine polysaccharides, it seems that some of them were able to decrease the perceived bitterness. Vidal and colleagues studied the effect of two fractions of wine polysaccharides (MPs + AGPs and RG II) on several sensory attributes of solutions of grape seed procyanidins [129]. The authors observed that the proteoglycan fraction (MPs + AGPs) was able to reduce bitterness, whereas RG II had no effect. The effect of CMC and arabic gum on the bitterness of gourd juice were also evaluated [164]. The authors showed that both polysaccharides increased the viscosity of the solution; however, only the addition of 5% arabic gum significantly reduced the bitterness of the bitter gourd juice [164].

## 4. Final Remarks

It is undeniable that tannins and their interaction with different food macromolecules, namely proteins and polysaccharides, and with salivary proteins are linked to astringency and bitterness. As thoroughly revised here, the interaction of tannins with (food or biological) proteins has been studied at a molecular level, providing extensive knowledge on the critical structural features of both tannins and proteins, the mechanisms underlying the interactions, as well as the effect of other physicochemical parameters such as the pH, temperature, and other solvents, such as ethanol. Regarding astringency perception, the current point of view is a simultaneous and dynamic contribution of the referred proposed mechanisms. However, concrete data about some of these mechanisms are yet to be obtained. Regarding bitterness, there is also a significant advance in the identification of bitter polyphenols, mainly from tannins group, although there is a lack of a knowledge on the structure-bitterness relationship.

It is also unquestionable that some polysaccharides are able to decrease both (salivary) protein-tannin interactions and the bitterness of several food products. The effect on protein-tannin interaction is widely demonstrated and supported by several research works. However, regarding the effect on bitterness modulation, there are few available data. Furthermore, sensory analysis is the main technique used to evaluate the perceived astringency upon addition of polysaccharides. Therefore, understanding the molecular mechanisms as well as the key structural features that confer the modulation ability of polysaccharides is quite difficult. Future works should study the effect of polysaccharides on bitterness by different approaches, and study the effect of polysaccharides on other mechanisms that explain astringency.

Future works should be focused on the development of more complex models, including different oral elements, to understand the dynamic contribution of each one to the overall interactions. Furthermore, these studies should be applied to mixtures of polyphenols instead of pure compounds. Finally, the experimental approaches should be adapted to be more similar to what occurs in the oral cavity during food intake.

## Figures and Tables

**Table 1:** Summary of studies on human bitter taste receptors (TAS2Rs) screening for activation by polyphenol agonists. EC_50_-Half-maximum effective concentration.

Hydrolyzable Tannins	Grandinin		Castalagin		Punicalagin			Vescalagin			PGG	
Structure	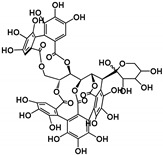		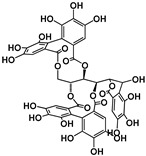		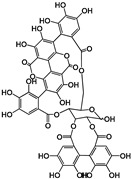			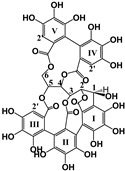			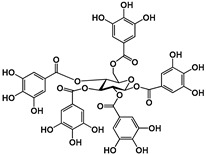	
EC_50_ (μM)	2.43		4.44		40.23; 3.95			7.26			8.5; 6.6	
Activated TAS2Rs	7		7		5 and 7			7			5 and 7	
Transfected cells	HEK293T											
Reference	[30]										[29]	
Condensed Tannins	(−)-Epicatechin	(+)-Catechin	ECG	EGCG		EGC	Procyanidins					
							B1		B4	B2g	B7	C2
Structure	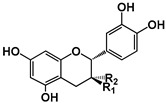 (−)-Epicatechin R1 = H, R2 = OH (−)-Catechin R1 = OH, R2 = H		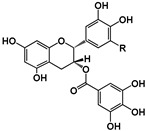 (−)-ECG R = H (−)-EGCG R = OH			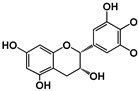	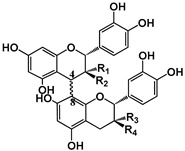 B1: R_1_ = OH; R_2_ = H; R_3_ = H; R_4_ = OH B4: R_1_ = H; R_2_ = OH; R_3_ = OH; R_4_ = H B2g: R_1_ = O-Galloyl; R_2_, R_3_ = H; R_4_ = OH				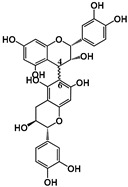	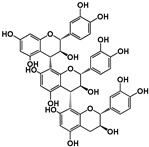
EC_50_ (μM)	30151.0; 3210.0; nd; 3800	nd; nd	70; nd; nd; 151	nd; 12.30; 34; nd; 8.50-161; 16.72		nd; nd	119.34; 123.95		nd	6.29; 9.11	nd	35.6
Activated TAS2Rs	4, 5, 14, 39	14 and 39	14, 30, 38, 39	4, 5, 14, 30, 39, 43		14 and 39	5 and 7		5	5 and 39	5	5
Transfected cells	HEK293 and HEK293T	HEK293	HEK293 and HEK293T				HEK293T					
Reference	[29,31,33]	[33]	[31,32,33]	[30,31,33]		[31,32,33]	[30]					[29]

nd: non determined; ECG: epicatechin gallate, EGCG: epigallocatechin gallate; EGC: epigallocatechin.

**Table 2:** Summary of the relevant studies on the interaction of different polysaccharides and tannins (binary system) and the effect of polysaccharides on the interaction between (salivary) proteins and tannins (ternary system).

**Binary System**				
	**Tannin**	**Polysaccharide**	**Techniques**	**Ref.**
	(+)-catechin	β-cyclodextrin	NMR; Molecular modelling	[165]
	(+)-catechin; (−)-epicatechin	β-cyclodextrin	NMR	[135]
	Apple procyanidins	Apple/citrus pectins	ITC	[141,142,143]
	Grape seed procyanidins	Wine polysaccharides	DLS	[158]
	Procyanidins	Apple/citrus pectins	UV-vis spectrophotometry	[142,143]
	Proanthocyanidins	Insoluble cell-wall material (CWM)	Phloroglucinolysis and SEC	[166]
	Apple procyanidins	Arabinan-rich pectic polysaccharides	ITC; UV-vis spectrophotometry	[167]
**Ternary System**				
**Protein**	**Tannin**	**Polysaccharide**	**Techniques**	**Ref.**
Caffeine (similar features with proteins)	Methylgallate and Trigalloylglucose	α- and β-cyclodextrin	NMR; ITC	[134]
Proline-rich gelatin; Sodium caseinate	PGG	Pectin; galactomannans; carrageenans	NMR	[132]
BSA	Grape seed procyanidins	Pectin; xanthan; polygalacturonic acid; Arabic gum; β-cyclodextrin; arabinogalactan; dextran; glucose	Nephelometry	[131,140]
BSA	Grape seed procyanidins	Xanthan; Arabic gum; dextran	Flow Nephelometry	[144]
α-amylase; IB8c	Grape seed procyanidins	AGPs; RG II	Light scattering	[151]
α-amylase	Grape seed procyanidins	Pectin; Arabic gum; cyclodextrin	DLS; nephelometry and fluorescence quenching	[145]
Salivary proteins	Grape seed procyanidins	Pectin; Arabic gum; polygalacturonic acid	HPLC; SDS-PAGE	[146]
Saliva	Proanthocyanidins	Wine oligosaccharides and polysaccharides (PRAGs; RG II; MPs)	Sensory analysis	[152]
aPRPs and P-B peptide	Procyanidin B2 and Punicalagin	RG II and AGPs	HPLC; Nephelometry; Fluorescence quenching	[153]
BSA	Wine tannins	Wine polysaccharides (AGPs; MPs; RG II)	HPLC-DAD; UV-vis spectrophotometry	[157]
BSA	Wine flavan-3-ols	Wine polysaccharide mixture	CD; SDS-PAGE; Fluorescence spectroscopy	[63]
Salivary proteins	Procyanidin B2 and Punicalagin	RG II and AGPs	HPLC; Nephelometry; Fluorescence quenching; SDS-PAGE	[154]
Salivary proteins	Grape seed procyanidins	Grape pectic polysaccharides	HPLC; SDS-PAGE;	[155]
Salivary proteins	Grape seed procyanidins	Commercial yeast mannoproteins	ITC; Molecular Dynamics Simulation	[156]

BSA: bovine serum albumin; aPRPs: acidic PRPs; NMR: nuclear magnetic resonance; ITC: isothermal calorimetry; DLS: dynamic light scattering; SEC: size exclusion chromatography; HPLC: high pressure liquid chromatography; SDS-PAGE: sodium dodecylsulphate polyacrylamide gel electrophoresis; CD: circular dichroism.
